# Mapping the multimodal connectome: On the architects of brain network science

**DOI:** 10.1371/journal.pbio.3002043

**Published:** 2023-03-06

**Authors:** Guusje Collin, Susan Whitfield-Gabrieli

**Affiliations:** 1 Department of Psychiatry, Radboud University Medical Center, Nijmegen, the Netherlands; 2 Donders Institute for Brain, Cognition and Behaviour, Nijmegen, the Netherlands; 3 Massachusetts Institute of Technology, McGovern Institute for Brain Research, Cambridge, Massachusetts, United States of America; 4 Department of Psychology, Northeastern University, Boston, Massachusetts, United States of America; 5 Department of Psychiatry, MGH, Harvard Medical School, Boston, Massachusetts, United States of America

## Abstract

Delineating the human brain network and analyzing its architecture is one of the major goals of modern neuroscience. This Perspective looks at a landmark PLOS Biology study from 2008 on structural connectome mapping and gauge how it shaped the field of brain network science.

## Teaser

Delineating the human brain network and analyzing its architecture is one of the major goals of modern neuroscience. Here, we commemorate a 2008 landmark structural connectome study in *PLOS Biology* and gauge how it shaped the field of brain network science.

This article is part of the *PLOS Biology* 20th Anniversary Collection.

All of our (cognitive) behavior requires the exchange and integration of neural information across spatially separated areas of the brain. This exchange of neural information among brain regions is facilitated and structured by the complex architecture of the brain’s connectional anatomy, which encompasses roughly 86 billion neurons organized into a network of local circuits that are interconnected by long-range axonal pathways.

Neuroscientists have long aspired to map this network. In 1665, Danish bishop and anatomist Niels Stensen (Nicolaus Steno) argued that to truly dissect white matter (which he referred to as “the great masterpiece of nature”) we would need “to trace the nervous filaments through the substance of the brain, to see which way they pass, and where they end” [1]. However, the first full description of all connections in a neural system took until 1986 to be completed. This neural network of the 1 mm-long roundworm *Caenorhabditis elegans* containing 302 neurons and approximately 7,000 connections is the only complete connectome of an adult organism to date. In recent years, however, synaptic-level connectomes have also been completed for the larva of the sea squirt *Ciona intestinalis*, the larva of the marine annalid *Platynereis dumerilii*, and the larva of the fruit fly, *Drosophila melanogaster* [2].

**Fig 1:**
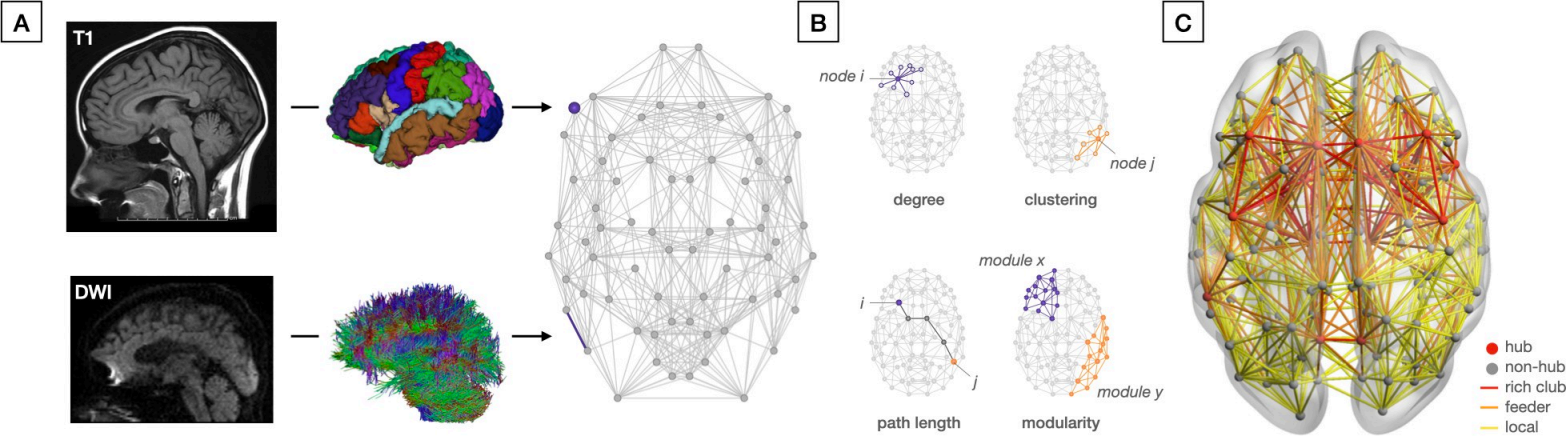
Connectome reconstruction and analysis. (A) Schematic depiction of connectome reconstruction with network nodes (i.e., brain regions) and edges (i.e., connections) derived from anatomical T1-weigthed and diffusion-weighted MRI data respectively. (B) Four graph metrics commonly used to assess the topological organization of a (brain) network, including: (1) degree, representing the number of connections of each node *i*; (2) clustering, which is a measure of the extent to which nodes in a network tend to cluster together (i.e., how many triangles any node *j* is part of); (3) path length, which reflects the average number of steps it takes to get from any node *i* to any node *j* in the network (as a measure of network efficiency); and (4) modularity, which measures the extent to which a network can be decomposed into modules (illustrated here module *x* and *y*). (C) Connectome reconstruction from neuroimaging data showing a “rich club” organization with brain hubs and interconnecting rich club connections in red, “feeder” connections between hubs and non-hubs in orange, and “local” connections between non-hubs in yellow. Created using BrainNet Viewer [13].

The desire to map the human brain network is inspired by the notion that delineation and analysis of its architecture may help us understand the working of the brain and its disorders [3], following the idea that “structure drives behavior”. This holds true from the cellular level, where the proteome (the entire set of proteins expressed by a genome, cell, or organism) drives cell behavior [4], to the social level, where office lay-out determines who we are friends with at work. It is thought to be similarly true for the brain. Although the vast size and complexity of the human brain prevents the reconstruction of the human brain network at the synaptic level currently and in the foreseeable future, advances in neuroimaging do allow an increasingly detailed reconstruction of the human connectome at the level of local brain regions and interconnecting fiber bundles (Fig 1).

In 2005, Olaf Sporns and Patric Hagmann, simultaneously but independently, coined the terms “connectome” as “a comprehensive structural description of the network of elements and connections forming the human brain” [5] and “connectomics” as the study of the brain’s set of structural connections [6]. The scientists subsequently teamed up to publish their seminal article “Mapping the Structural Core of the Human Cerebral Cortex” in *PLOS Biology* in 2008 [7]. In this study, the research teams used diffusion spectrum imaging to map structural brain networks of individual subjects. Using graph theory, these connectomes were analyzed in terms of network organization and found to contain a structural core in posterior medial and parietal cortex constituting highly connected and highly central connector hubs that interlinked the structural modules in the network. With resting-state fMRI from the same subjects, they further showed that the strength of these structural connections estimated from diffusion imaging was highly correlated with the strength of positive functional connections seeded from posterior medial cortex [7], suggesting that structural brain connections shape the brain’s functional topology, particularly of the then recently discovered default mode network [8]. Although the study was performed with data from just 5 individual subjects, all key results have since been confirmed by several independent studies, illustrating the robustness of their findings.

Subsequent studies benefited from advances in brain parcellation schemes, particularly those grounded in cortical anatomy, which have helped integrate human brain connectivity and architecture. Moreover, developments in graph analysis have advanced our understanding of brain network organization, showing for example that the connectome is organized according to a “rich club” topology, i.e., encompassing a central club of highly connected brain hubs [9]. Likened to a kind of G8-club of the brain, this central hub system is even more strongly interconnected than can be explained by the hubs’ high connectivity alone and appears to form an essential part of “the structural backbone of brain communication” delineated by Hagmann and colleagues/Sporns. Papers like the one by Hagmann and Sporns have also inspired the undertaking of the Human Connectome Project, which is now the main benchmark and data source for this kind of research. In addition, they contributed to the even more ambitious endeavor of reconstructing the whole brain at the level of synaptic connectivity. In the field of “micro-connectomics,” which is now slowly coming of age, the current focus is on establishing the ultrastructural connectivity of the whole mouse brain.

Hagmann and Sporns’ seminal work [7] continues to potentiate methodologically innovative investigations of structural and functional connectomics, including studies of structure–function relationships. Despite the prevailing notion that brain function is likely constrained by its underlying structure and anatomy, several studies on structure–function coupling have shown relatively modest associations between structural and functional MRI data. Both methodological and biological factors may contribute to these findings. In terms of methodology, although diffusion MRI has been a major step forward in estimating structural connectivity in vivo, limitations include the occurrence of false negatives and false-positive connections and a lack of directionality and laminar specificity. Functional MRI suffers from different, but equally substantial, limitations. Novel approaches, however, including the application of deep learning to create personalized predictions of functional connectivity from an individual’s structural connectome do show high prediction accuracies and explain significant inter-individual variation in cognitive performance [10]. In terms of biology, structural and functional networks may not align uniformly across the brain. Studies using biophysical models now show that brain structure and function may be closely aligned in unimodal cortex (i.e., primary sensorimotor regions), but appear to uncouple gradually in higher-order transmodal cortex (particularly in default mode and salience networks) [11]. In all, these recent findings suggest that structure–function relations may indeed be much tighter than suggested in earlier studies and also more complex according to regional variation in functional and cytoarchitectonic cortical hierarchies.

Going forward, the hope is that the field of connectomics will help elucidate the pathophysiology of brain disorders and contribute towards the development of novel biomarkers for prediction and treatment. For example, elucidating white matter tracts activated by deep brain stimulation (DBS) targets may provide critical information about the circuit substrates mediating DBS efficacy in mitigating treatment-resistant depression. In addition, connectome-based predictive modeling coupled with experience sampling may be employed to predict fluctuations in mental features such as mind wandering or clinical symptoms within an individual over time [12]. These promising new directions ultimately build upon Hagmann and Sporns’ pioneering work, and the field owes a debt of gratitude to their efforts.
