# Correction: Mapping the multimodal connectome: On the architects of brain network science

**DOI:** 10.1371/journal.pbio.3002219

**Published:** 2023-07-13

**Authors:** Guusje Collin, Susan Whitfield-Gabrieli

There is an error in reference 2. The correct reference is: Winding M, Pedigo BD, Barnes CL, Patsolic HG, Park Y, Kazimiers T, et al. The connectome of an insect brain. Science. 2023; 379(6636). https://www.science.org/doi/10.1126/science.add9330
